# Circulating apelin levels fail to link sarcopenia-related muscle parameters in older adults

**DOI:** 10.1016/j.jnha.2024.100475

**Published:** 2025-01-08

**Authors:** Eunhye Ji, So Jeong Park, Il-Young Jang, Ji Yeon Baek, Yunju Jo, Hee-Won Jung, Eunju Lee, Dongryeol Ryu, Beom-Jun Kim

**Affiliations:** a Asan Institute for Life Sciences, Asan Medical Center, University of Ulsan College of Medicine, Seoul 05505, South Korea; b Division of Geriatrics, Department of Internal Medicine, Asan Medical Center, University of Ulsan College of Medicine, Seoul 05505, South Korea; c Department of Biomedical Science and Engineering, Gwangju Institute of Science and Technology, Gwangju 61005, South Korea; d Division of Endocrinology and Metabolism, Department of Internal Medicine, Asan Medical Center, University of Ulsan College of Medicine, Seoul 05505, South Korea

**Keywords:** Apelin, Sarcopenia, Biomarker, Older adults, Aging, Skeletal muscle

## Abstract

**Background:**

Based on the compelling experimental evidence supporting apelin’s beneficial effects on muscle metabolism, our study aimed to evaluate the role of circulating apelin levels as a biomarker for muscle health in humans.

**Methods:**

This investigation employed a cross-sectional design, encompassing 237 community-dwelling older adults aged ≥65 years who underwent comprehensive geriatric evaluations in South Korea. Sarcopenia diagnosis was based on Asian-specific criteria, and serum apelin concentrations were determined using enzyme immunoassay techniques.

**Results:**

Following adjustment for potential confounding factors, no significant disparities in serum apelin levels were observed between sarcopenic and non-sarcopenic individuals, nor were differences detected based on skeletal muscle mass, strength, or physical performance categories (*P* = 0.335 to 0.765). Furthermore, circulating apelin concentrations showed no significant correlations with any sarcopenia assessment metrics, including skeletal muscle index, grip strength, gait speed, chair stand test duration, or short physical performance battery score (*P* = 0.170 to 0.832). Elevations in serum apelin levels were not significantly associated with the risk of sarcopenia or compromised muscle phenotypes (*P* = 0.452 to 0.896). Additionally, stratification of participants into quartiles based on serum apelin concentrations revealed no significant variations in sarcopenia-related parameters across groups (*P* = 0.197 to 0.592).

**Conclusion:**

These findings suggest that, contrary to previous studies in cellular and animal models where apelin demonstrated a protective impact on muscle homeostasis, such effects may not translate to the human context, and contribute valuable clinical evidence indicating that serum apelin may not serve as a reliable biomarker for sarcopenia.

## 1 Introduction

Sarcopenia, characterized by the progressive loss of skeletal muscle mass and function, has emerged as a critical public health concern, particularly among the aging population [[1], [2], [3]]. Its clinical significance extends beyond its direct impact on physical performance and quality of life, as it is also associated with a spectrum of adverse health outcomes, including an increased risk of falls, frailty, hospitalization, and mortality [[4], [5], [6]]. Despite the growing recognition of sarcopenia as a major contributor to morbidity and the healthcare burden, early diagnosis and effective management remain challenging due to the condition's complex and multifactorial nature [7]. This highlights the urgent need for reliable biomarkers that can facilitate early detection, risk stratification, and monitoring of treatment efficacy. The discovery of novel biomarkers for sarcopenia would not only deepen our understanding of its underlying pathophysiological mechanisms but also pave the way for personalized interventions and targeted therapies, ultimately improving patient outcomes and alleviating the socioeconomic burden associated with this age-related muscle disorder.

Apelin, a multifunctional endogenous peptide, interacts with its G protein-coupled receptor (APJ) to regulate a broad spectrum of physiological processes [8]. This versatile molecule plays critical roles in cardiovascular function, neuroprotection, immune responses, and glucose homeostasis [[9], [10], [11]]. Increasing research evidence has highlighted apelin as a potential target for anti-aging interventions, particularly concerning muscle health and metabolism. Notably, the expression of apelin and its receptor APJ declines with age, correlating with deteriorating muscle function in rodent models [12,13]. Moreover, apelin-deficient mice exhibit accelerated aging phenotypes, including the premature onset of sarcopenia [12,13]. Conversely, apelin administration has been shown to enhance muscle strength and physical activity in older mice, effectively reversing age-related sarcopenia and restoring youthful behavioral patterns and circadian rhythms [12]. Despite compelling experimental evidence supporting apelin's beneficial effects on muscle metabolism, the role of circulating apelin levels in relation to muscle health in humans remains controversial [[13], [14], [15], [16], [17], [18]]. Accordingly, our study aimed to evaluate the utility of circulating apelin as a biomarker for sarcopenia by measuring serum apelin levels and conducting comprehensive assessments of muscle phenotypes—muscle mass, strength, and physical performance—which are essential for diagnosing sarcopenia in a cohort of older adults.

## 2 Materials and methods

### 2.1 Study participants

This clinical research targeted Korean individuals aged 65 and above who underwent comprehensive geriatric assessments at the Division of Geriatrics or Endocrinology within the Department of Internal Medicine at Asan Medical Center (AMC) in Seoul, Korea, between October 2020 and October 2021. Participants attended the clinic due to non-specific symptoms such as fatigue and appetite loss, which are prevalent among older adults, or for managing chronic conditions like osteoarthritis, hypertension, and hyperlipidemia. All participants were community-dwelling and mobile, not residing in nursing homes or hospitals. The study excluded individuals with end-stage renal disease, active cancers, or symptomatic heart failure with a life expectancy under one year. Blood samples for assessing sarcopenia were collected from 237 eligible participants during their visit, following the acquisition of informed consent and the exclusion of those not meeting the criteria. The AMC Institutional Review Board approved the study (approval number 2021-0155), ensuring compliance with the ethical principles of the Declaration of Helsinki.

### 2.2 Evaluation of sarcopenia

Trained nurses collected demographic data and medical histories through interviews and reviews of medical records. Body composition, including muscle mass, was assessed using bioelectrical impedance analysis (BIA) with the InBody S10 device (InBody, Seoul, South Korea), which utilizes multiple frequencies (1, 5, 50, 250, 500, and 1000 kHz). The appendicular skeletal muscle mass (ASM), representing the total limb muscle mass, was calculated. The skeletal muscle mass index (SMI) was derived by dividing ASM by the square of the participant’s height (kg/m²). Muscle strength was measured by assessing handgrip strength in the dominant hand using a hand dynamometer (Patterson Medical, Warrenville, IL, USA). Participants were seated with elbows bent at 90 degrees and instructed to apply maximum force on the dynamometer; two attempts were made with a one-minute interval, and the highest value was recorded. Gait speed was measured over a four-meter distance, and the time taken to complete five chair stands was recorded. Additionally, the short physical performance battery (SPPB), which includes chair stands, balance tests, and gait speed assessment, was administered.

Sarcopenia diagnosis was based on the 2019 Consensus Guidelines from the Asian Working Group for Sarcopenia [3]. Participants were diagnosed with sarcopenia if they exhibited low muscle mass along with either reduced muscle strength or impaired physical performance. Low muscle mass was defined as an SMI below 7.0 kg/m² for men and below 5.7 kg/m² for women. Muscle strength was considered weak if handgrip strength was under 28 kg for men and 18 kg for women. Poor physical performance was indicated by a gait speed of less than 1.0 m/s, taking more than 12 seconds for five chair stands, or an SPPB score of 9 or lower.

### 2.3 Measurement of serum apelin levels

Blood samples were drawn from the antecubital vein in the morning after participants fasted overnight for at least 8 h. Samples were centrifuged at 3000 rpm for 5 min at 4 °C, and the supernatant was separated to remove cell debris. Samples with clotting or hemolysis were excluded from analysis. Serum samples were stored at −80 °C until apelin levels were measured. Apelin concentrations were determined using a previously validated nonselective apelin-12 enzyme immunoassay kit that recognizes the C-terminal sequence of 12 amino acids shared among all apelin isoforms (Phoenix Pharmaceuticals, Belmont, CA). The kit had a detection limit of 0.07 ng/mL, with intra-assay and inter-assay coefficients of variation below 10% and 15%, respectively.

### 2.4 Sample size estimation

The current study included 237 community-dwelling older adults aged 65 years and older. This sample size exceeds the numbers reported in comparable studies investigating the relationship between circulating apelin levels and aging-related health outcomes. Specifically, a study by Jang et al. [18] explored serum apelin levels and their association with frailty-related functional parameters in 80 older adults, divided into robust, prefrail, and frail groups. This analysis, which relied on phenotypic and deficit-accumulation frailty indices, concluded that this sample was sufficient for evaluating differences in serum apelin levels across these groups after adjusting for key confounders. Given the similarities in methodological approaches, including the use of serum apelin levels as a primary biomarker and comprehensive assessments of aging-related parameters, our study's larger cohort provides increased statistical power to detect meaningful associations. Furthermore, a previous study estimated that a minimum of 23 participants per group would suffice for two-group comparisons, assuming a significance level of 0.05 and a power of 0.80​. With 57 sarcopenic and 180 non-sarcopenic participants in our study, the sample size far exceeds this threshold, allowing robust subgroup analyses and ensuring sufficient representation across the spectrum of sarcopenia-related muscle phenotypes.

### 2.5 Statistical analysis

Data are presented as means ± standard deviation (SD) for continuous variables, and as frequencies and percentages for categorical variables. We compared the baseline characteristics of participants with and without sarcopenia using Student's t-test for continuous variables and the chi-square test for categorical variables. Analysis of covariance (ANCOVA) was employed to evaluate the adjusted means of serum apelin levels based on sarcopenia status and associated parameters, with adjustments for potential confounders. The confounding variables, including sex, age, fat mass, diabetes, cardiovascular disease, and regular exercise, were selected based on their significant association with apelin in the univariate analysis or their clinical relevance. Regular exercise was defined as engaging in moderate-intensity physical activities characterized by slightly increased breathing at least three times per week. Linear regression analyses were utilized to explore the relationships between serum apelin levels and muscle parameters pertinent to sarcopenia, both unadjusted and adjusted for confounders. In these analyses, serum apelin levels were log-transformed due to their skewed distribution (Supplementary Fig. S1). Logistic regression was conducted to estimate the odds ratios for the risk of sarcopenia and adverse muscle outcomes with each SD increase in serum apelin levels. Furthermore, ANCOVA was used to compare the adjusted means of sarcopenia parameters across quartiles of serum apelin levels. All statistical analyses were performed using SPSS version 18.0 (SPSS Inc., Chicago, IL, USA), with a two-sided *P* value of less than 0.05 considered significant.

## 3 Results

The baseline characteristics of the 237 study participants are presented in Table 1. Among the participants, 57 (24.1%) were classified as having sarcopenia, while 180 (75.9%) were non-sarcopenic. The proportion of females in the sarcopenic and non-sarcopenic groups was 51 (89.5%) and 134 (74.4%), respectively (*P* = 0.017). Participants with sarcopenia were significantly older (78.6 ± 6.4 years) compared to those without sarcopenia (73.3 ± 6.5 years) (*P* < 0.001). Compared to the control group, individuals with sarcopenia exhibited lower body weight, height, BMI, SMI, grip strength, gait speed, and SPPB scores, and required more time to complete the chair stand test (all *P* < 0.001).

**Table 1:** Baseline characteristics of the study participants according to their sarcopenia status.

Variables	Sarcopenia (n = 57)	No sarcopenia (n = 180)	*P* value
Sex, n (%)			**0.017**
Female	**51 (89.5)**	**134 (74.4)**	
Male	**6 (10.5)**	**46 (25.6)**	
Age, years	**78.6 ± 6.4**	**73.3 ± 6.5**	**<0.001**
Weight, kg	**52.9 ± 8.4**	**61.1 ± 11.3**	**<0.001**
Height, cm	**150.9 ± 6.4**	**156.6 ± 7.8**	**<0.001**
BMI, kg/m^2^	**23.2 ± 3.4**	**24.8 ± 3.7**	**<0.001**
SMI, kg/m^2^	**5.16 ± 0.53**	**6.43 ± 1.11**	**<0.001**
Grip strength, kg	**19.0 ± 4.9**	**25.9 ± 8.2**	**<0.001**
Gait speed, m/s	**0.78 ± 0.25**	**1.07 ± 0.23**	**<0.001**
Chair stand, s	**17.0 ± 13.0**	**10.1 ± 5.5**	**<0.001**
SPPB score (range, 0–12)	**8.98 ± 2.73**	**11.11 ± 1.80**	**<0.001**
Fat mass, kg	20.7 ± 6.6	21.6 ± 7.2	0.393
Diabetes, n (%)	18 (31.6)	67 (37.2)	0.527
Cardiovascular disease, n (%)	**13 (22.8)**	**15 (8.3)**	**0.008**
Regular exercise, n (%)	8 (14.0)	32 (17.8)	0.685

The data are reported as mean ± standard deviation, unless otherwise indicated. Values achieving statistical significance are highlighted in bold text. Continuous variables were compared between the two groups using the Student's t-test, and categorical variables were assessed with the chi-square test. BMI, body mass index; SMI, skeletal muscle mass index; SPPB, short physical performance battery.

**Fig. 1:**
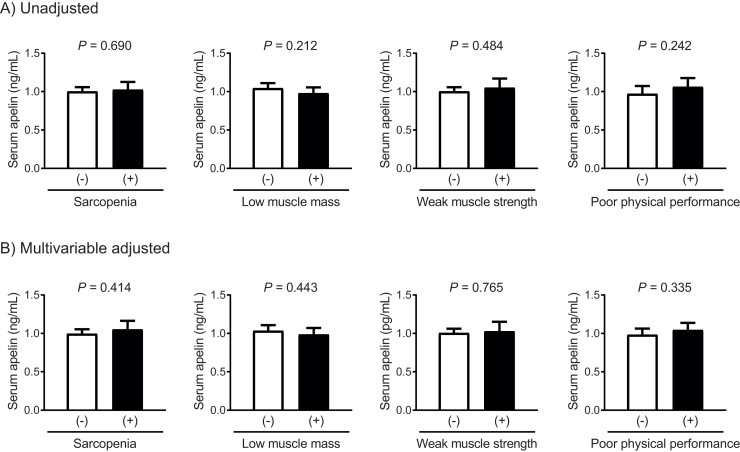
Serum apelin levels relative to sarcopenia status and associated parameters. (A) Unadjusted analysis. (B) Multivariable adjusted analysis. Multivariable adjustment model includes sex, age, fat mass, diabetes, cardiovascular disease, and regular exercise. The estimated means along with their 95% confidence intervals were calculated and compared using analysis of covariance.

The mean, median, SD, and range of serum apelin levels were 1.01 ng/mL, 1.11 ng/mL, 0.59 ng/mL, and 0.09–2.90 ng/mL, respectively. Differences in serum apelin levels according to the presence of sarcopenia and associated parameters were analyzed using ANCOVA (Fig. 1). However, no significant differences in serum apelin levels were observed based on the presence of sarcopenia, low muscle mass, weak muscle strength, or poor physical performance, either before or after adjustment for sex, age, fat mass, diabetes, cardiovascular disease, and regular exercise (*P* = 0.212 to 0.765).

The association between circulating apelin levels and muscle phenotypes required for diagnosing sarcopenia was examined using linear regression analysis (Table 2). In the unadjusted model, serum apelin levels were inversely associated with SMI, grip strength, and gait speed (*P* < 0.001 to 0.023), but no significant correlations were observed with other muscle parameters (*P* = 0.582 to 0.755). However, after adjusting for sex, age, fat mass, diabetes, cardiovascular disease, and regular exercise, serum apelin levels did not show significant associations with SMI, grip strength, gait speed, time to complete the chair stand test, or SPPB score (*P* = 0.170 to 0.832). Similarly, in logistic regression analysis, neither before nor after adjustment for confounding variables did an increase of 1 SD in serum apelin levels show a significant difference in the odds ratios for sarcopenia, low muscle mass, weak muscle strength, or poor physical performance (Table 3; *P* = 0.215 to 0.896).

**Table 2:** Linear regression analyses to determine the association between serum apelin levels and sarcopenia parameters.

	Unadjusted				Multivariable adjusted			
	β	SE	*β*	*P*	β	SE	*β*	*P*
SMI	**–0.586**	**0.085**	**–0.411**	**<0.001**	–0.157	0.114	–0.110	0.170
Grip strength	**–3.504**	**0.615**	**–0.349**	**<0.001**	–0.326	0.875	–0.032	0.710
Gait speed	**–0.049**	**0.021**	**–0.148**	**0.023**	–0.042	0.042	–0.128	0.315
Chair stand test	0.380	0.691	0.036	0.582	–0.295	1.386	–0.028	0.832
SPPB score	0.057	0.184	0.020	0.755	–0.291	0.350	–0.104	0.407

The Enter method was applied to this model. Serum apelin levels were log-transformed due to their skewed distribution. Multivariable adjustment model includes sex, age, fat mass, diabetes, cardiovascular disease, and regular exercise. Values achieving statistical significance are highlighted in bold text. β, unstandardized regression coefficient; SE, standard error; *β*, standardized regression coefficient; SMI, skeletal muscle mass index; SPPB, short physical performance battery.

**Table 3:** Logistic regression analyses to determine the odds ratios for sarcopenia and the related parameters according to the increase in serum apelin levels.

	Unadjusted		Multivariable adjusted	
	Odds ratio (95% CI) per SD increment in serum apelin	*P* value	Odds ratio (95% CI) per SD increment in serum apelin	*P* value
Sarcopenia	1.10 (0.70−1.73)	0.689	1.18 (0.69−2.03)	0.546
Low muscle mass	0.75 (0.48−1.18)	0.215	0.85 (0.51−1.42)	0.541
Weak muscle strength	1.19 (0.73−1.85)	0.484	1.04 (0.56−1.94)	0.896
Poor physical performance	1.17 (0.90−1.51)	0.242	0.89 (0.65−1.22)	0.452

Multivariable adjustment model includes sex, age, fat mass, diabetes, cardiovascular disease, and regular exercise. CI, confidence interval; SD, standard deviation.

**Fig. 2:**
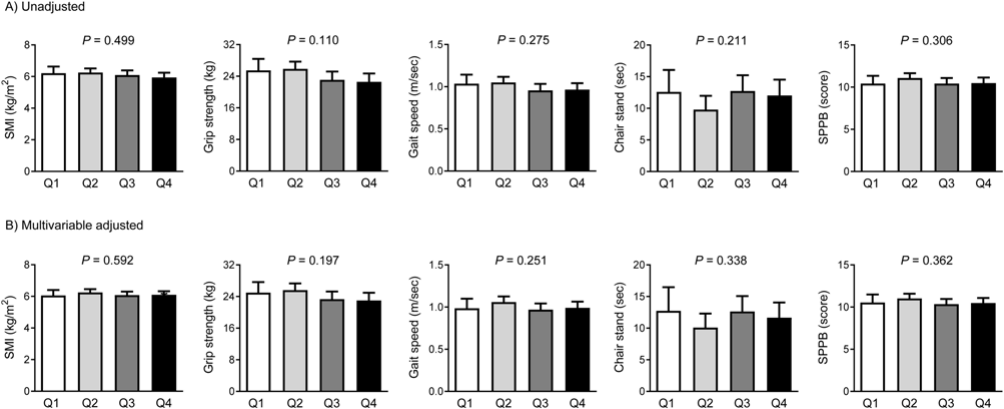
Differences in sarcopenia components based on serum apelin quartiles. (A) Unadjusted analysis. (B) Multivariable adjusted analysis. Multivariable adjustment model includes sex, age, fat mass, diabetes, cardiovascular disease, and regular exercise. The estimated means along with their 95% confidence intervals were calculated and compared using analysis of covariance. Serum apelin quartiles: Q1 = 0.09–0.37 ng/mL; Q2 = 0.38–1.10 ng/mL; Q3 = 1.11–1.34 ng/mL; Q4 = 1.35–2.90 ng/mL.

To assess whether circulating apelin levels influence human muscle phenotypes beyond specific thresholds, participants were divided into four groups based on serum apelin concentrations (Fig. 2). However, no significant differences were observed in SMI, grip strength, gait speed, time to complete the chair stand test, or SPPB score across serum apelin quartiles, both before and after adjusting for sex, age, fat mass, diabetes, cardiovascular disease, and regular exercise (*P* = 0.110 to 0.592).

## 4 Discussion

In this cohort study involving ambulatory, community-dwelling older adults, we found no significant differences in serum apelin concentrations between individuals with and without sarcopenia or poor muscle phenotype. Furthermore, linear regression analysis revealed no correlations between serum apelin levels and muscle parameters used to define sarcopenia. These findings suggest that, contrary to earlier studies in cellular and animal models where apelin demonstrated a beneficial impact on muscle metabolism, such effects may not translate to the human context, and contribute valuable clinical evidence indicating that serum apelin may not serve as a reliable biomarker for sarcopenia.

In recent years, apelin has emerged as a promising therapeutic agent for attenuating sarcopenia, with multiple mechanisms of action identified in cellular and animal studies. Apelin stimulates mitochondrial biogenesis through the upregulation of PGC-1α, enhancing energy production and metabolic function in muscle tissues [19,20]. Additionally, apelin promotes protein synthesis by activating the AMPK and PI3K/Akt signaling pathway, leading to muscle hypertrophy and the preservation of muscle mass [13,[21], [22], [23]]. The regulation of autophagy is another critical function of apelin, as it enhances the clearance of damaged organelles and proteins, thereby maintaining muscle integrity [13,24]. Apelin also exerts anti-inflammatory effects by reducing the levels of pro-inflammatory cytokines, such as TNF-α and IL-6 [13,25], which are associated with muscle degradation. Furthermore, apelin boosts muscle regenerative capacity by promoting satellite cell proliferation and activation [13,26], essential for muscle repair. As an exercise-induced myokine, apelin's production is typically elevated following physical activity, but it declines in aging skeletal muscle, correlating with reduced muscle function [12,13]. Preclinical studies demonstrate that apelin reverse sarcopenia through enhancing muscle mass, fiber hypertrophy, and functional parameters [12,13]. Collectively, these mechanisms underline the potential of apelin as a multifaceted approach to counteract age-associated muscle wasting.

Apelin has garnered significant attention not only as a potential therapeutic target for sarcopenia due to its mechanistic properties but also as a candidate biomarker, given its measurable presence in blood as a secreted factor. This dual potential has prompted several clinical investigations. Initial studies showed promise, demonstrating a positive correlation between exercise-induced elevations in plasma apelin concentrations and SPPB scores [13]. However, subsequent investigations failed to establish a significant association between serum apelin levels and skeletal muscle mass or other muscular parameters in human participants [14,16]. As a result, current evidence does not support the conclusive use of circulating apelin as a biomarker for sarcopenia. It is important to note that previous studies were limited in their ability to draw robust conclusions due to relatively small sample sizes, focus on specific populations such as elderly malnourished patients or professional soccer players, or insufficient muscle assessments that did not meet international diagnostic criteria for sarcopenia [[13], [14], [15],^18^]. Our study addresses these limitations by comprehensively evaluating all aspects of muscle parameters, including muscle mass, muscle strength, and various physical performance measures in a large cohort. Furthermore, our primary outcome is based on international criteria with race-specific cutoffs, providing methodological advantages over previous investigations. Consequently, despite apelin's favorable effects on muscle observed in experimental studies, our research does not substantiate the utility of blood apelin concentration as a biomarker for sarcopenia in adults aged 65 years and older.

The absence of a significant association between circulating apelin levels and muscle phenotypes in humans could be attributed to several complex and interrelated factors. First, sarcopenia is a multifaceted condition influenced by a wide array of determinants, including genetic predisposition, physical activity levels, nutritional status, hormonal changes, and chronic inflammation [1,3]. These factors collectively contribute to muscle mass and function, potentially overshadowing the role of apelin as a single biomarker. Second, the synthesis of apelin begins with a 77-residue preproapelin, which undergoes cleavage into a 55-residue proapelin and further into active isoforms between 13 and 36 residues [27]. Various isoforms, including apelin-13, apelin-17, and apelin-36, are likely present in biological fluids such as blood [[27], [28], [29], [30], [31]] and may play distinct roles in muscle physiology and sarcopenia. It is plausible that measuring specific apelin isoforms in blood could yield results different from ours. This hypothesis highlights the need for future studies employing isoform-specific assays or localized measurements, such as muscle biopsies, to better elucidate the relationship between apelin and muscle health. Third, while this study measured circulating apelin concentrations, these levels do not necessarily reflect the localized muscle environment. A potential mismatch between apelin's systemic and localized roles in muscle physiology may contribute to the observed lack of association. To date, no studies have directly compared systemic apelin concentrations with apelin levels in muscle tissue. Investigating this aspect further could provide critical insights into the mechanisms underlying sarcopenia and its biomarkers. Fourth, the complexity of APJ receptor expression, including its distribution and sensitivity, may vary significantly among individuals [32,33], further complicating the detection of a clear association between apelin levels and muscle phenotypes. Finally, in vitro studies and animal models often involve controlled environments that do not fully replicate the complexity of human physiology. Experimental conditions, such as the dosage and administration of apelin in preclinical settings, may differ substantially from the natural fluctuations of apelin in humans. These differences could lead to findings that do not translate directly to human conditions. Collectively, these factors underscore the need for further investigation into the role of apelin in human muscle physiology, with particular focus on the distinctions between systemic and localized effects, isoform-specific dynamics, and methodological standardization in future studies.

When interpreting the findings of our study, several limitations must be taken into account. First, the cross-sectional design of this research allows us to identify associations between variables, but it does not permit the determination of causal effects. Additionally, the study exclusively involved individuals aged 65 and older from South Korea, which limits the applicability of our results to other ethnicities and age groups. Furthermore, the selection bias associated with sampling from an outpatient clinic at a single hospital may arise from the inclusion of individuals presenting with symptoms such as fatigue, appetite loss, or chronic conditions, potentially introducing confounding factors that could influence the observed relationship between apelin and sarcopenia. Lastly, there remains the possibility circulating apelin levels or muscle metabolism in humans may be affected by uncontrolled factors, which could have impacted our findings.

In conclusion, our study demonstrates that serum apelin concentrations did not exhibit significant associations with any muscle phenotypes, including skeletal muscle mass, grip strength, gait speed, chair stand test performance, and SPPB score, in a cohort of older adults where sarcopenia was accurately assessed according to international criteria. While these findings do not negate the beneficial effects of apelin on muscle metabolism observed in experimental studies, they suggest that such results may not directly translate to human populations. Our data indicate that although apelin may remain a potential therapeutic target for sarcopenia, circulating apelin levels may not serve as an optimal biomarker for reflecting muscle health in humans. To establish the utility of serum apelin as a predictive marker for sarcopenia, additional large-scale longitudinal studies and repeated measurements over time are required to account for the dynamic nature of apelin levels and their fluctuations due to acute or chronic factors.

## Funding

This research was supported by grants from the Korean ARPA-H Project through the Korea Health Industry Development Institute (KHIDI), funded by the Ministry of Health and Welfare, Republic of Korea [Grant Number: RS-2024-00507256]; the Korea Health Technology R&D Project through KHIDI, also funded by the Ministry of Health and Welfare, Republic of Korea [Grant Number: RS-2024-00401934]; the Asan Institute for Life Sciences, Asan Medical Center, Seoul, Republic of Korea [Grant Number: 2024IL0015]; and the National Research Foundation of Korea (NRF), funded by the South Korean government (MSIT) [Grant Number: 2021R1C1C2006842].

## Data availability

The data that support the findings of this study are available from the corresponding author by investigators with institutional review board approval.

## Declaration of competing interest

The authors have nothing to disclose.
