# Supplementary material for: Circulating apelin levels fail to link sarcopenia-related muscle parameters in older adults
Source: J Nutr Health Aging. 2025 Jan 8;29(3):100475. doi: 10.1016/j.jnha.2024.100475 (PMC12179996; doi:10.1016/j.jnha.2024.100475)
Supplement: Supplementary file 1 [file mmc1.docx]

**Supplementary Fig. 1.** Serum apelin distribution.
